# Detection of influenza-like illness aberrations by directly monitoring Pearson residuals of fitted negative binomial regression models

**DOI:** 10.1186/s12889-015-1500-4

**Published:** 2015-02-21

**Authors:** Ta-Chien Chan, Yung-Chu Teng, Jing-Shiang Hwang

**Affiliations:** Research Center for Humanities and Social Sciences, Academia Sinica, 128 Academia Road, Section 2, 115 Nankang, Taipei, Taiwan; Institute of Statistical Science, Academia Sinica, 128 Academia Road, Section 2, 115 Nankang, Taipei, Taiwan

**Keywords:** Influenza surveillance, Negative binomial model, CUSUM, Pearson residual, Outpatient, Emergency department

## Abstract

**Background:**

Emerging novel influenza outbreaks have increasingly been a threat to the public and a major concern of public health departments. Real-time data in seamless surveillance systems such as health insurance claims data for influenza-like illnesses (ILI) are ready for analysis, making it highly desirable to develop practical techniques to analyze such readymade data for outbreak detection so that the public can receive timely influenza epidemic warnings. This study proposes a simple and effective approach to analyze area-based health insurance claims data including outpatient and emergency department (ED) visits for early detection of any aberrations of ILI.

**Methods:**

The health insurance claims data during 2004–2009 from a national health insurance research database were used for developing early detection methods. The proposed approach fitted the daily new ILI visits and monitored the Pearson residuals directly for aberration detection. First, negative binomial regression was used for both outpatient and ED visits to adjust for potentially influential factors such as holidays, weekends, seasons, temporal dependence and temperature. Second, if the Pearson residuals exceeded 1.96, aberration signals were issued. The empirical validation of the model was done in 2008 and 2009. In addition, we designed a simulation study to compare the time of outbreak detection, non-detection probability and false alarm rate between the proposed method and modified CUSUM.

**Results:**

The model successfully detected the aberrations of 2009 pandemic (H1N1) influenza virus in northern, central and southern Taiwan. The proposed approach was more sensitive in identifying aberrations in ED visits than those in outpatient visits. Simulation studies demonstrated that the proposed approach could detect the aberrations earlier, and with lower non-detection probability and mean false alarm rate in detecting aberrations compared to modified CUSUM methods.

**Conclusions:**

The proposed simple approach was able to filter out temporal trends, adjust for temperature, and issue warning signals for the first wave of the influenza epidemic in a timely and accurate manner.

**Electronic supplementary material:**

The online version of this article (doi:10.1186/s12889-015-1500-4) contains supplementary material, which is available to authorized users.

## Background

Novel influenza viruses such as the 2009 pandemic H1N1 influenza [1,2] and 2013 H7N9 influenza outbreaks in China [3] have made the public aware of the threat of influenza infection. In fact, seasonal influenza epidemics have occurred annually and caused heavy disease burdens and high economic losses around the world [4,5]. Although vaccination among children [6] and the elderly [7] has been proven to be beneficial for preventing some infections and reducing the severity of influenza outbreaks, most adults are still exposed to the threat of influenza, especially for novel influenzas [8]. In order to perform public health intervention such as vaccination and health education, and to understand the epidemic trends in communities, many types of traditional public health surveillance such as sentinel physician surveillance [9] and virological surveillance [10] have been implemented. After the September 11 attacks in 2001, the United States began to develop emergency department (ED)-based syndromic surveillance systems for detecting any aberrations of syndromes and diseases [11,12]. The timeliness and efficiency are improved over the traditional surveillance systems.

The best approach to disease surveillance is to create a seamless surveillance system without extra labor involved in reporting. Symptom-based surveillance is one example which automatically aggregates either specific International Classification of Diseases (ICD) codes [13] or chief complaints [14] from medical records. In addition, health insurance claims data are the other important source recording patients’ diagnosis based on the ICD codes. In Taiwan, the coverage rate of national health insurance (NHI) was over 98% in 2010 [15]. It would be a great advantage to utilize the daily series of influenza-like illness (ILI) outpatient and ED visits in communities for outbreak detection in local areas.

Currently, there are many available statistical methods for detecting aberrations in influenza surveillance, including the seasonal regression model [16], time series [17], Bayesian model [18], modified cumulative sum (CUSUM) [19], adaptive CUSUM (ACUSUM) [20], optimal exponentially-weighted moving average (EWMA) [21] and SaTScan space-time permutation model [22]. However, previous published systems have focused mainly on the daily or weekly total ILI visits aggregated in a large area, without considering repeat clinical visits during the same infection course, which might mask the true epidemic trend of ILI incidence. To account for this situation, we first defined a relatively small study area including a major district and several neighboring districts, and proposed using only the first occurrence of influenza clinical visits in the study area within 14 days for the patients for further daily updating of the model. Considering geographical differences and various weather patterns in northern, central and southern Taiwan, we select one study area from each of the three regions. Since many environmental or systematic factors such as temperature, relative humidity [23], national holidays, and day of the week are believed to be associated with influenza epidemics and clinical visits, we proposed a simple and effective approach by first adjusting the effects of these deterministic and potentially confounding factors from the daily series of medical visits in a study area with a negative binomial regression model to take account of over-dispersion, and then monitoring the standardized Pearson residuals directly for aberration detection. We evaluated the performance of the proposed method by comparing the issued warning signals with virological surveillance during the 2009 pandemic (H1N1) influenza period in Taiwan. We also conducted a simulation study to compare the performance between the proposed approach and modified CUSUM method.

## Methods

### Study overview

The proposed approach for detecting the aberrations of ILI outbreaks in a study area consists of two stages. In the first stage, we used negative binomial regression models to fit daily outpatient and ED ILI visits during 2004–2007, and selected significant predictors for the three study areas in northern, central and southern Taiwan, separately. The residual of observed number of visits on the last day of each observed series of visits was further standardized. In the second stage, we monitored these daily standardized Pearson residuals for any aberrations in 2008 and 2009. Then, we evaluated the performance of the proposed approach by the empirical health insurance claims data in 2009, when novel H1N1 pandemic flu outbreaks occurred. The detected aberrations were compared with the weekly influenza virus isolation rates. In addition, we compared the widely used surveillance method, modified CUSUM methods applied to both observed visits and Pearson residuals, with the proposed approach using simulated data.

### Data source

**Figure 1:**
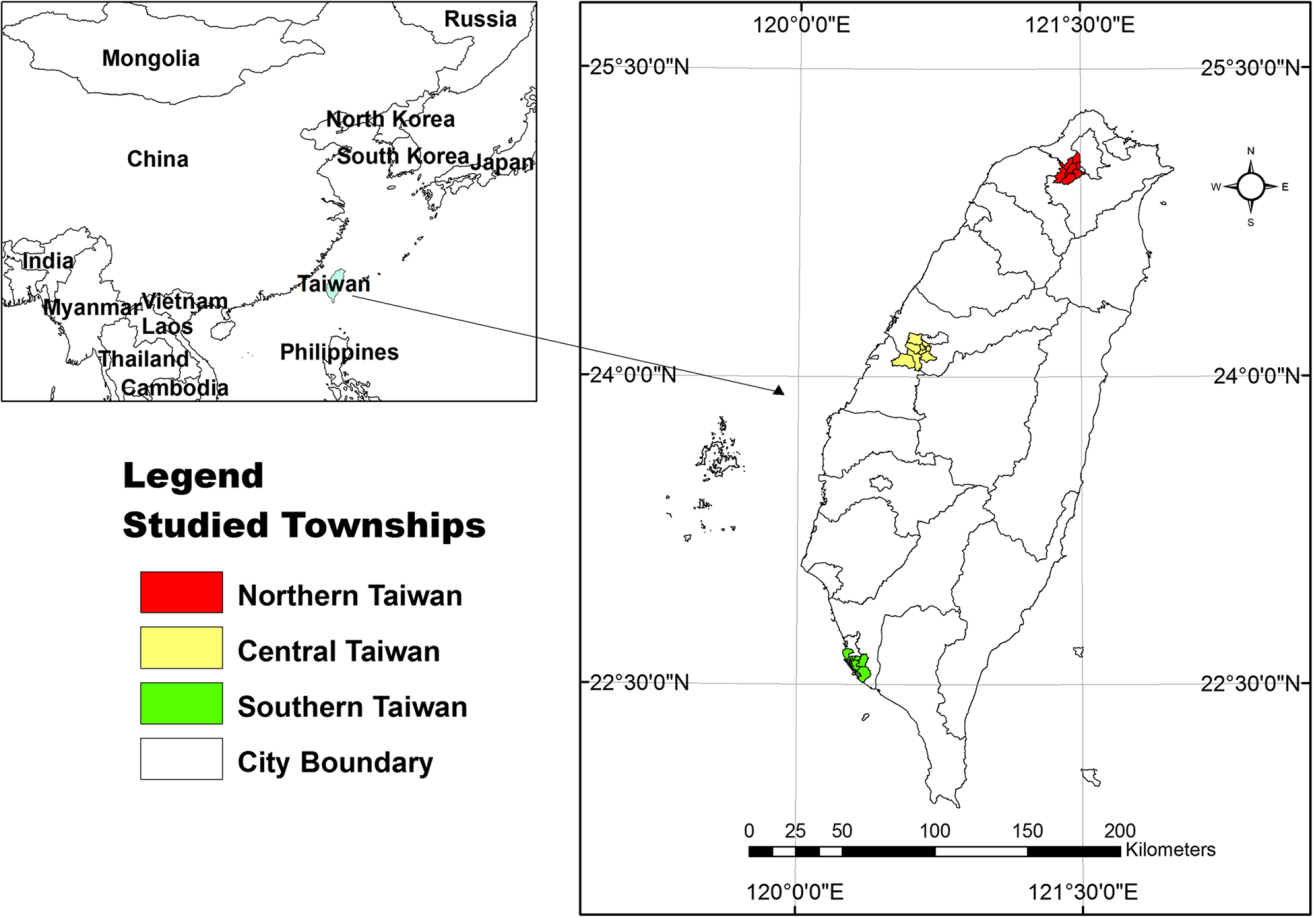
**Three selected study areas in Taiwan.**

The three study areas selected in this study are shown on the map (Figure 1). Each study area consists of several populous districts surrounding a major weather station. The registered residents of the three study areas in northern, central and southern Taiwan in 2009 numbered 2,356,205, 1,330,913 and 1,120,944, respectively. Daily ILI visits from 2004 to 2009 were obtained from the NHI research database of the National Health Research Institute. This study was approved by the institutional review board (IRB) of Academia Sinica (IRB#: AS-IRB01-12117). The database we used was all stripped of identifying information, and thus informed consent was not needed. Daily meteorological data were downloaded from the Data Bank for Atmospheric Research maintained by National Applied Research Laboratories (http://dbar.ttfri.narl.org.tw). Weekly influenza virus isolation rates were calculated from a laboratory surveillance database maintained by Taiwan’s CDC.

### ILI definition and the first occurrence of ILI cases

The NHI research database, also called the hospital-based health insurance claims database, is publicly available to researchers in Taiwan. There are four major levels of the health care system in Taiwan, including medical centers (Level 4), regional hospitals (Level 3), area hospitals (Level 2) and primary health care (i.e. clinics, Level 1). In order to focus on the influenza epidemics at the community level, we restrict the data of outpatient and ED visits to the lower levels of the health care system. For the outpatient visits, only area hospitals and primary health care were included. For the ED visits, only regional hospitals and area hospitals were included.

The ILI cases in this study were determined as those diagnosed with 29 ILI-related ICD-9 codes, which were the definition in the ESSENCE system (Electronic Surveillance System for the Early Notification of Community-based Epidemics, United States) [24,25]. In this study, we proposed analyzing daily series of new ILI cases. If a case had repeated ILI visits within 14 days, only the first visit was counted. The daily series of new ILI cases was easily created because, in the NHI research database, each patient has a scrambled unique identification code and dates of clinical visits. The reason for using 14 days as an observational window was that the influenza incubation period is about 1–4 days, and the virus shedding occurs from the day before symptoms begin through 5–10 days after illness onset [26]. The sum of these two periods has a maximum of around 14 days. Therefore, we defined multiple ILI visits within 14 days as being in the same infection course, and counted only the first occurrence.

### Negative binomial regression model and application to empirical data

For the daily counts of outpatient or ED ILI visits in a study area, we proposed use of a negative binomial regression model to better account for over-dispersion, which was observed in our exploratory data analysis. We utilized the “glm.nb” function under open-source software, an R [27] package named “MASS”, to implement the negative binomial regression [28]. The regression model for estimating expected visits of the last day of the observed series was constructed by fitting the daily series of visits and covariates observed in the three years before the last day. In practice, the regression models needed to be updated for each day and each study area. To simplify evaluation of the method, we first used the data of the years 2004–2007 in the three study areas to identify influential covariates for the regression models. The models with the selected covariates were then repeatedly fitted to daily ILI visits observed each day during 2008–2009 and three years before that day.

During the variable selection stage, the significance level was set at 0.05. The weather factor used here was whether the temperature was equal to or below 14°C, which is the official definition of a continental cold air mass by the Central Weather Bureau in Taiwan (http://www.cwb.gov.tw/V7e/knowledge/encyclopedia/me003.htm). In addition, the temporal dependence was also considered by including the new ILI cases observed exactly one week earlier. Our exploratory data analyses showed day of the week, Chinese New Year, national holidays, typhoon days off, the day following national holidays and typhoon days off may have influences on the observed visits. They are mainly related to the closure of the hospitals and clinics. The seasonality of influenza epidemic has often been modeled with harmonic terms. We have found that these terms of sine and cosine functions sometimes were not good enough to capture the seasonal pattern. In this study, we proposed an alternative seasonal term called moving month-of-the-year time-dependent variable, which is the difference between medians of visits in the past 30 days and visits in the past 365 days for seasonal adjustment in the model. This time-dependent variable is determined dynamically by the observed data. It can be used for adjusting usual season outbreaks. If a factor or variable was statistically significant in an area, it was selected into the regression model as a covariate. Chinese New Year and national holidays, including national public holidays of Taiwan and typhoon days off, were obtained from the Directorate-General of Personnel Administration, Executive Yuan, Taiwan (http://www.dgpa.gov.tw/). The days of the week had clear effects on the clinic visits; for example, there were very small numbers of outpatient visits on Sundays, and more outpatient visits on Mondays and ED visits on Saturdays and Sundays, while both outpatient and ED visits were stable from Tuesday to Friday. Once the covariates were fixed, we used the repeatedly fitted negative binomial regression models to estimate the expected visits for each day in 2008 and 2009 in each area.

Specifically, for estimating expected number of visits on day *t*, we have observed daily ILI outpatient or ED visits and the vector of selected covariates, denoted by *y*_*i*_ and *X*_*i*_ for *i* = *t*, *t* − 1, …, *t* − *T*_0_. We fixed *T*_0_ = 365 × 3 in the real data analysis. The mean and variance of *y*_*i*_ for the negative binomial model are denoted as *E*(*y*_*i*_) = *μ*_*i*_ and $Var\left({y}_i\right)={\mu}_i+\kappa {\mu}_i^2$, where the constant *κ* is called a dispersion parameter. The mean equation of the negative binomial regression model is often given by ${\mu}_i= \exp \left({X}_i^T\beta \right)$, where *β* is a vector of coefficients. Let ${\widehat{\beta}}_t$ and ${\widehat{\kappa}}_t$ be the estimated vector of coefficients and dispersion parameter from the fitted model using the series of *y*_*i*_ and *X*_*i*_ for *i* = *t* − 1, …, *t* − *T*_0_. The expected number of visits for day *t* is estimated by ${\widehat{y}}_t= \exp \left({X}_t^T{\widehat{\beta}}_t\right)$. The variance of estimate is given by $Var\left({\widehat{y}}_t\right)={\widehat{y}}_t+{\widehat{\kappa}}_t{\widehat{y}}_t^2$. The Pearson residuals were denoted as ${R}_t=\left({y}_t-{\widehat{y}}_t\right)/\sqrt{Var\left({\widehat{y}}_t\right)}$ for *t* = 1, 2, …, *n*, from the fitted negative binomial regression models on *n* consecutive days.

### Aberration detection rule

Since the sample size *T*_0_ is often large, the standardized Pearson residuals could be assumed to be approximately distributed normally with mean 0 and variance 1. Therefore, we proposed a simple rule by directly monitoring the series of Pearson residuals. When a Pearson residual was larger than the 100(1 − *α*)^th^ percentile of the standard normal distribution, denoted by *z*_1 − *α*_, a signal was issued for the day to report a possible aberrant outbreak. We suggest choosing *α* = 0.025, that is, set the threshold *z*_0.975_ = 1.96 for simplicity. If the series of visits were well fitted by the negative binomial models, the false alarm rate would be around 2.5%. In practice, we should expect a false alarm rate slightly larger than 2.5% because the fitted models were usually not perfect. We may choose a smaller *α* if a false alarm rate is a major concern. However, the identification of an epidemic may be delayed or even undetected. For comparison with the CUSUM method in this study, we name our proposed approach SPR due to use of Standardized Pearson Residuals *R*_*t*_ as the deviation statistic for outbreak detection and threshold *z*_1 − *α*_.

### Simulation study

After applying the proposed approach to the empirical data, the performance of the approach was still hard to evaluate due to the unknown daily virus isolation in a small area. Thus, we designed a simulation study to mimic outbreaks and compared the performance of the proposed SPR approach with the popular modified CUSUM. First, time series of counts on *T* = 760 calendar days, denoted by *w*_1_, …, *w*_*T*_, were generated from negative binomial distributions with mean *μ*_*i*_ = exp(5 + 0.2*x*_1*i*_ + *x*_2*i*_) and dispersion parameter *κ*_*i*_ = 0.2/*μ*_*i*_ so that variance of *w*_*i*_ equals 1.2*μ*_*i*_ for the *i*^th^ day. The covariates *x*_1*i*_ and *x*_2*i*_ were generated to represent the seasonal pattern and day-of-the-week profile, respectively. The consecutive sets of 30 elements (assumed to be 30 days per month) *x*_1*i*_ were generated from normal distributions with means of 2, 2, 2, 1, 0, −1, −2, −2,-2, −1, 0, and 1 for the 12 months, and standard deviation 0.1, to reflect more visits in winter and fewer visits in summer. The 7 elements of *x*_2*i*_ were generated from normal distributions with means 0.1, 2, 1.5, 1.5, 1.5, 1.5, and 1 and standard deviation 0.1 for each week repeatedly to mimic the much fewer visits on Sunday and more on Monday observed from the empirical data.

We then assumed there is an epidemic period starting on day 601 and lasting for 40 days. The daily new cases were determined by ${o}_i=\theta \times sd\left({w}_i\right)\times \exp \left[1-\frac{{\left(i-621\right)}^2}{400}\right]$ for the epidemic period, where *θ* is a fixed parameter of signal-to-noise ratio, and $sd\left({w}_i\right)=\sqrt{1.2{\mu}_i}$ is the standard deviation of *w*_*i*_. The final number of visits *y*_*i*_ is *w*_*i*_ plus the integer part of *o*_*i*_ for the epidemic period, and *w*_*i*_ for the other regular days.

We considered three signal-to-noise ratios *θ* = 1, 3, 5. To get an insight of how strong the signals appear in the generated data, we give three daily series of visits simulated from the models with the three signal-to-noise ratios in Additional file 1, Additional file 2, Additional file 3. The patterns of the simulated daily visits look like what we might observe in the real world. The signals were hardly seen in the series of counts with weak signal-to-noise ratio *θ* = 1 but were very clear with *θ* = 5. We then used the models to simulate 1000 data sets for each *θ* value. For each data set, negative binomial regression models were fitted to a series of *T*_0_ = 360 days before each day starting from the 361^th^ to the 760^th^ day. The model included an intercept and 11 dummy variables representing months 2 to 12 and 6 dummy variables for Monday to Saturday as covariates. Note that we have simply used 11 dummy variables for describing seasonal pattern, which are not the true seasonal pattern used for generating the observations. This is to mimic the fact that we often don’t have perfect models in practice. We considered two thresholds with *α* = 0.025 and *α* = 0.005 in the simulation study with expectation of false alarm rates a little higher than 2.5% and 0.5%, respectively. For performance evaluation, we defined a measure of days to detection as the outbreak issuing day during the outbreak period minus 601, the true outbreak day. If no outbreak warning was issued during the epidemic period, event undetected is counted once. False alarm rate is defined as the percentage of signals issued on the remaining 360 regular days. We summarized the three performance measures from the 1000 simulations in the following. The mean days to detection is calculated by the average of days to detection on those simulated data for which all methods had successfully issued an alarm during the epidemic period. The probability of the epidemic going undetected is the number of times the epidemic was undetected divided by 1000. The mean false alarm rate is the average of the 1000 false alarm rates for each method.

The modified CUSUM method implemented in the Early Aberration Reporting System (EARS) is given by the following formula [29]. The deviation statistic is calculated on the observed counts and is denoted by ${C}_3(t)={\displaystyle \sum_{i=t}^{t-2} \max \left[0,{C}_2(i)-1\right]}$, where ${C}_2(i)=\frac{y_i-\overline{y}}{s}$, the sample mean $\overline{y}=\frac{1}{k}{\displaystyle \sum_{j=i-3}^{i-k-2}{y}_j}$, and sample variance ${s}^2=\frac{1}{k-1}{\displaystyle \sum_{j=i-3}^{i-k-2}{\left({y}_j-\overline{y}\right)}^2}$ for some *k* < (*t* − 3). We chose *k* = 7 as suggested in EARS. It signals on day *t* when the deviation statistic *C*_3_(*t*) is larger than a threshold, the value of which is often difficult to determine. It has been suggested that deviation statistic *C*_3_(*t*) was better calculated on model-based residuals. For a fair comparison in the simulation study, we also considered replacing original observations *y*_*i*_ with the Pearson residuals *R*_*i*_ from the same fitted negative binomial regression models in the above C3 algorithm. For the simulation study, thresholds of 1.28 and 2.88 were chosen for the CUSUM methods applied to observations and Pearson residuals, respectively, to keep the false alarm rates higher than those of the proposed SPR methods.

## Results

### Simulation data

The deviation statistic of SPR and CUSUM from days 361 to 760 are plotted along with three simulated data in the Additional file 1, Additional file 2, Additional file 3. From the plots, we can see the deviation statistics during the epidemic periods went up to cross the thresholds quickly when the signals were strong. We have also seen several false alarms issued on the regular days. The results of proposed SPR and CUSUM methods applied to three groups of 1000 simulated data sets with signal-to-noise ratios 1, 3 and 5 are summarized in Table 1. The proposed SPR with threshold *z*_1 − 0.025_ had the best performance in terms of early outbreak detection and very small probabilities of unsuccessful detection. The mean false alarm rate is about 3.6%, which is larger than the expected 2.5%, and smaller than the roughly 5% of the CUSUM methods. The SPR with threshold *z*_1 − 0.005_ could reduce the mean false alarm rate to about 1.1%, which is larger than the expected 0.5%. The cost of reducing the false alarm rate by about 2.5% was a long delay in early detection when the signal is weak. The results of CUSUM methods show that using Pearson residuals was indeed better than using the original observations in terms of detection power when the signals were not weak. When the signals were weak during the epidemic period, the CUSUM methods had very high probability of being unable to detect the epidemic, 0.37 and 0.18 for using residuals and observations, respectively, while the SPR method with threshold using *z*_1 − 0.025_ had a probability only 0.004 of failing to issue an alarm during the epidemic period. This simulation study demonstrated that the proposed SPR with a threshold of *z*_1 − 0.025_ or around 1.96 is a promising alternative approach in aberration detection.

**Table 1:** **Summary of simulation results**

**Signal to noise**	**Evaluation measure**	**Methods**			
		**SPR with threshold** ***z***_1 − 0.025_	**SPR with threshold** ***z***_1 − 0.005_	**CUSUM with pearson residuals**	**CUSUM with observations**
*θ* = 5	Mean days to detection	0.5	0.8	1.9	6.1
	Non-detection probability	0.0	0.0	0.013	0.082
	Mean false alarm rate	3.4%	1.0%	5.2%	5.2%
*θ* = 3	Mean days to detection	1.3	2.7	4.7	11.7
	Non-detection probability	0.0	0.0	0.105	0.158
	Mean false alarm rate	3.6%	1.1%	5.2%	5.2%
*θ* = 1	Mean days to detection	6.6	12.6	11.9	19.2
	Non-detection probability	0.004	0.120	0.373	0.178
	Mean false alarm rate	3.9%	1.2%	5.1%	5.3%

### Empirical data

From the fitted regression models on the daily series of ILI medical visits and covariates of the years 2004–2007, we determined the final covariates in three areas of Taiwan, listed in Table 2, for the regression models to be used for prediction in 2008 and 2009. In summary, outpatient ILI visits were high on Mondays and low on Sundays, Chinese New Year, other national holidays and typhoon days. In northern Taiwan and central Taiwan, cold temperature (≤14°C) was statistically significant (p < 0.05) and had positive correlation to ILI outpatient visits. The moving month-of-the-year variable and ILI visits on the day of the previous week were all slightly correlated with ILI visits in both ED and outpatient. In contrast, Saturdays, Sundays, Chinese New Year and other national holidays all positively correlated with ED ILI visits. Cold temperature was only statistically significant (p < 0.05) in northern Taiwan.

**Table 2:** **Selected explanatory variables for modeling influenza-like illness visits in different areas of Taiwan using daily visits in 2004 – 2007**

**Outpatient**			**Emergency department**	
Northern Taiwan				
Selected Variables	β	p-value	β	p-value
Monday	0.14	<0.01	0.12	<0.01
Saturday	-	-	0.23	<0.01
Sunday	−0.59	<0.01	0.95	<0.01
Chinese New Year	−1.66	<0.01	1.32	<0.01
Other national holidays	−0.35	<0.01	0.58	<0.01
Typhoon day	−0.89	<0.01	0.48	<0.01
After day off	0.17	0.01	-	-
Seasonal term	1.0 × 10^−3^	<0.01	0.07	<0.01
Temp. drop (≤14°C)	0.12	0.01	0.1	0.03
Visits a week ago	1.5 × 10^−3^	<0.01	0.05	<0.01
Central Taiwan				
Monday	0.16	<0.01	-	-
Saturday	-	-	0.23	<0.01
Sunday	−0.62	<0.01	0.67	<0.01
Chinese New Year	−1.29	<0.01	1.12	<0.01
Other national holidays	−0.24	<0.01	0.16	0.02
Typhoon day	−0.66	<0.01	-	-
Seasonal term	0.7 × 10^−3^	0.01	0.04	<0.01
Temp. drop (≤14°C)	0.13	0.02		
Visits a week ago	1.4 × 10^−3^	<0.01	0.04	<0.01
Southern Taiwan				
Monday	0.19	<0.01	0.08	<0.01
Saturday	-	-	0.21	<0.01
Sunday	−0.73	<0.01	0.86	<0.01
Chinese New Year	−1.18	<0.01	1.39	<0.01
Other national holidays	−0.32	<0.01	0.44	<0.01
Typhoon day	−0.83	<0.01	0.32	0.04
Seasonal term	2.2 × 10^−3^	<0.01	0.13	<0.01
Visits a week ago	1.0 × 10^−3^	<0.01	0.05	<0.01

**Figure 2:**
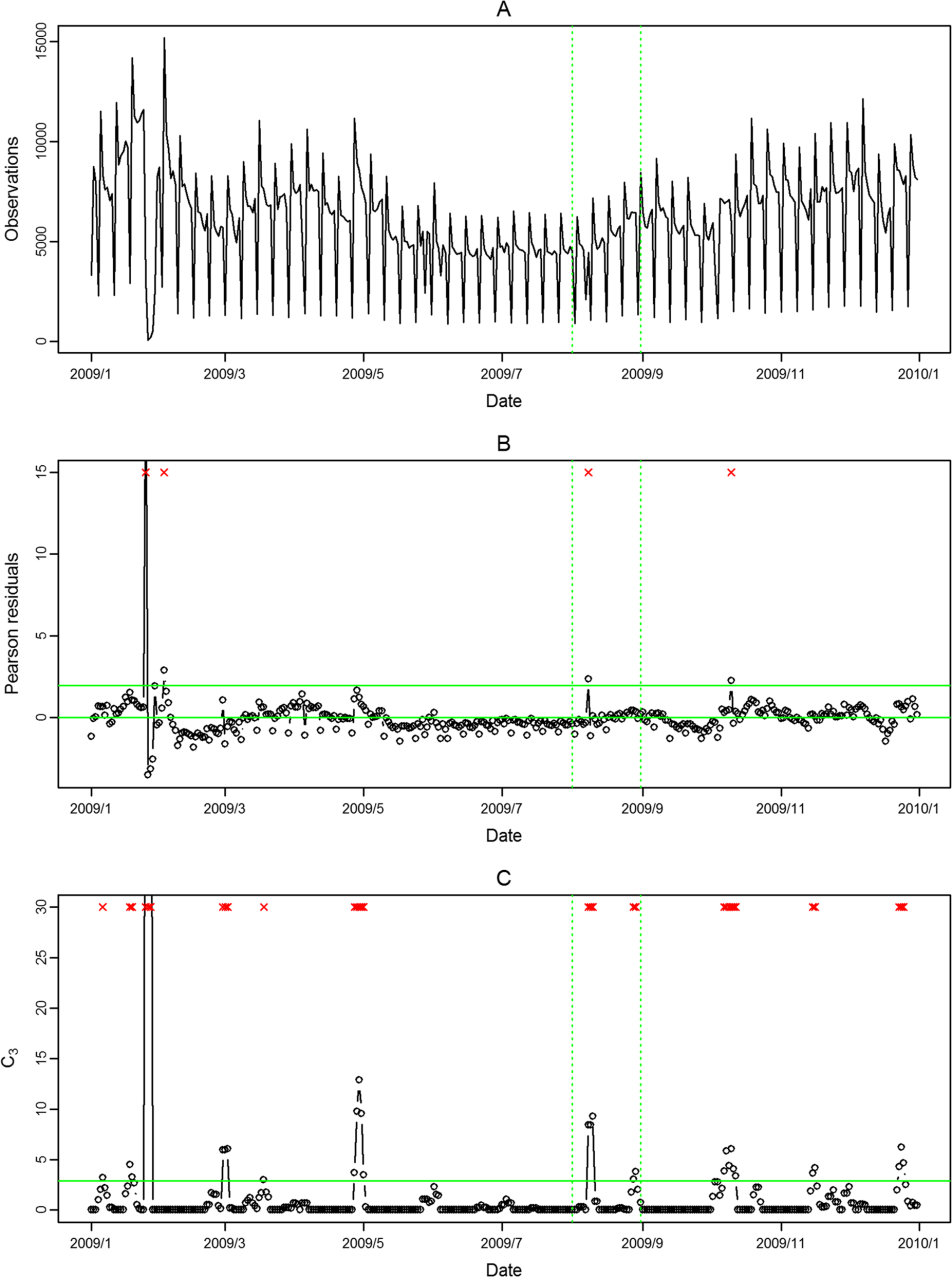
**Daily observed influenza-like illness outpatient visits in northern Taiwan in 2009 (A), and results of aberration detection by proposed method (B), by modified CUSUM applied to the Pearson residuals (C).** *Note: Detected aberration signals are marked with a red x at the top. The time period between the two dashed lines was August 2009.

**Figure 3:**
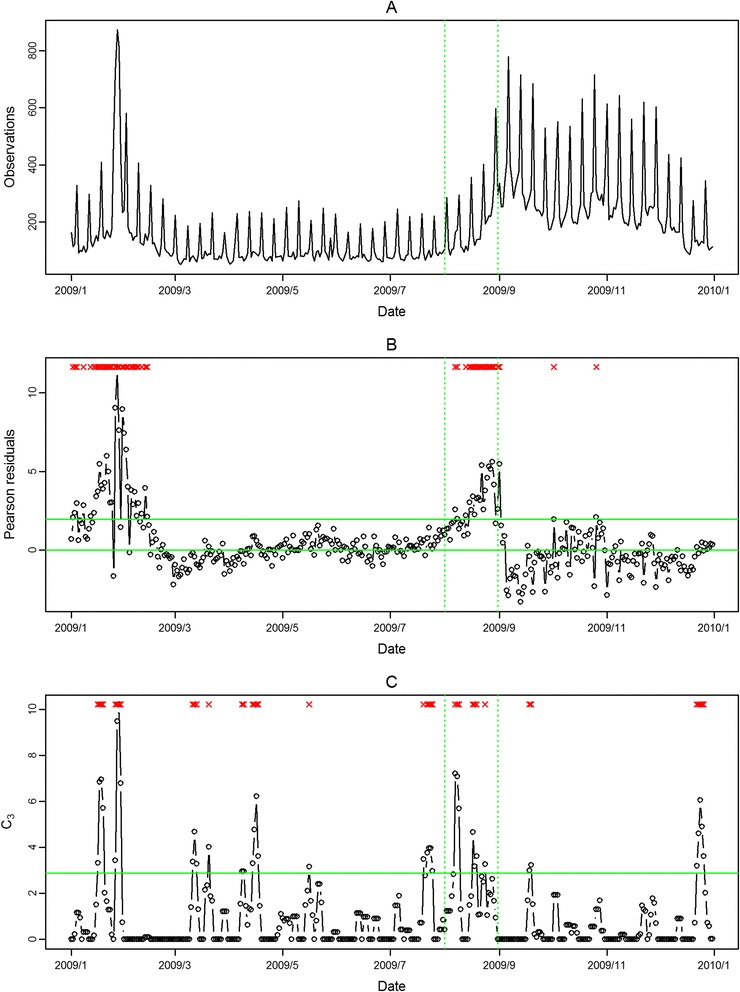
**Daily observed influenza-like illness emergency department visits in northern Taiwan in 2009 (A), and results of aberration detection by proposed method (B), by modified CUSUM applied to the Pearson residuals (C).** *Note: Detected aberration signals are marked with a red x at the top. The time period between the two dashed lines was August 2009.

The seasonal influenza epidemic in 2008 was mild and had no pandemic influenza outbreak. Thus, we evaluated the performance of the proposed approach in aberration detection only for 2009 in the following. In Figure 2 and Figure 3, the aberrations of seasonal influenza epidemic in February 2009 were detected in both outpatient and ED visits in northern Taiwan with our approach. However, the pandemic H1N1 influenza outbreaks in August 2009 were only detected in ED visits. The modified CUSUM method applied to the same Pearson residuals with a threshold 2.88 issued many false alarms in both outpatient and ED visits.

**Figure 4:**
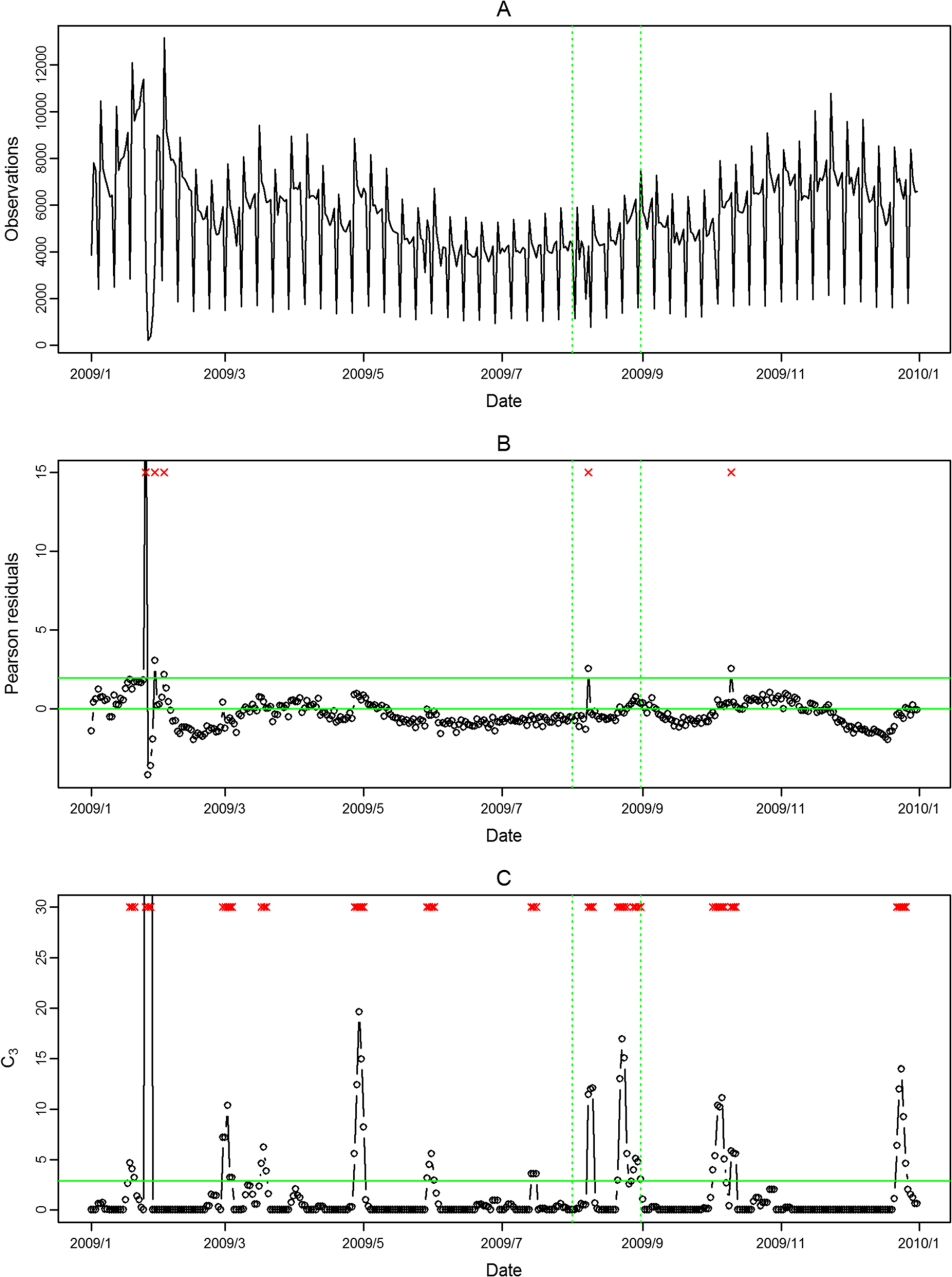
**Daily observed influenza-like illness outpatient visits in central Taiwan in 2009 (A), and results of aberration detection by proposed method (B), by modified CUSUM applied to the Pearson residuals (C).** *Note: Detected aberration signals are marked with a red x at the top. The time period between the two dashed lines was August 2009.

**Figure 5:**
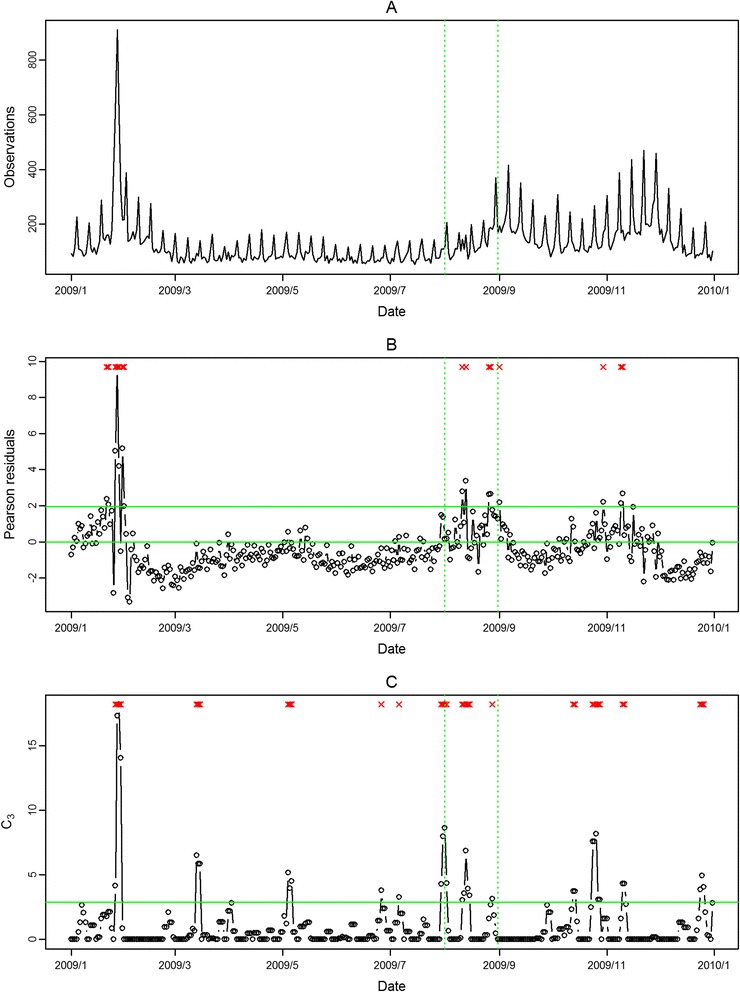
**Daily observed influenza-like illness emergency department visits in central Taiwan in 2009 (A), and results of aberration detection by proposed method (B), by modified CUSUM applied to the Pearson residuals (C).** *Note: Detected aberration signals are marked with a red x at the top. The time period between the two dashed lines was August 2009.

**Figure 6:**
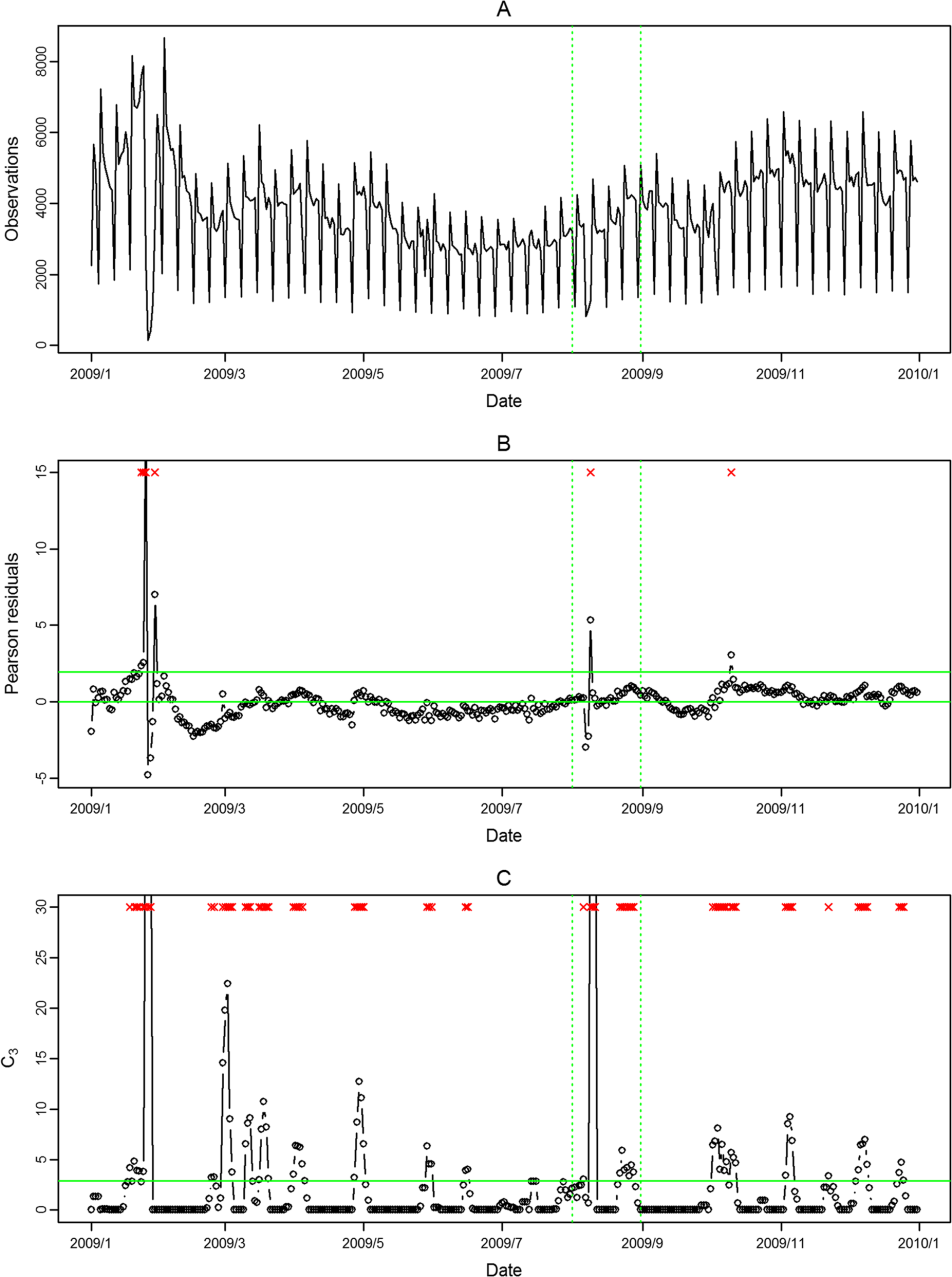
**Daily observed influenza-like illness outpatient visits in southern Taiwan in 2009 (A), and results of aberration detection by proposed method (B), by modified CUSUM applied to the Pearson residuals (C).** *Note: Detected aberration signals are marked with a red x at the top. The time period between the two dashed lines was August 2009.

**Figure 7:**
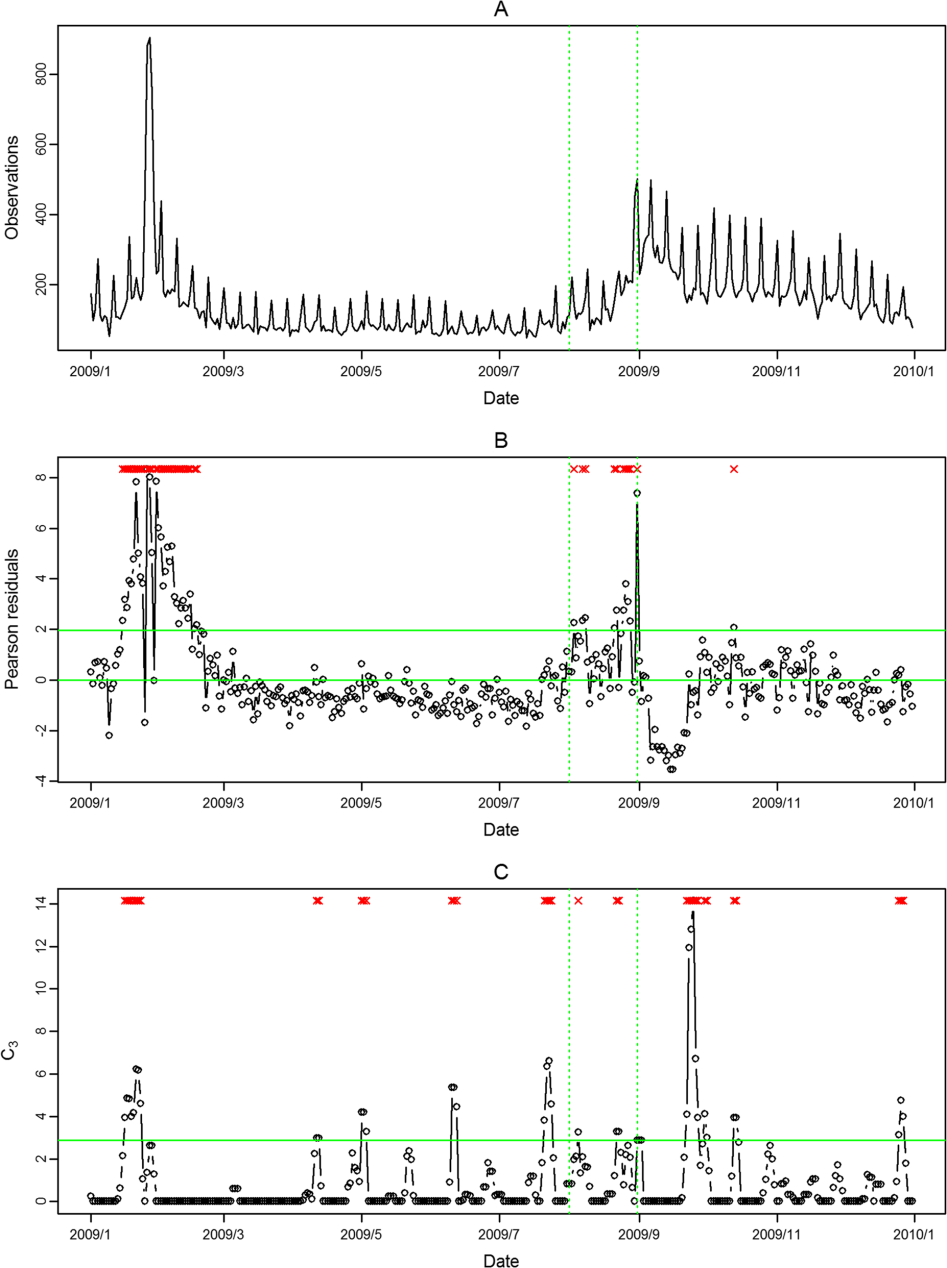
**Daily observed influenza-like illness emergency department visits in southern Taiwan in 2009 (A), and results of aberration detection by proposed method (B), by modified CUSUM applied to the Pearson residuals (C).** *Note: Detected aberration signals are marked with a red x at the top. The time period between the two dashed lines was August 2009.

In Figure 4, Figure 5, Figure 6, Figure 7, the seasonal influenza epidemics and the pandemic outbreaks were all detected in central and southern Taiwan with our approach. The intensities of the aberrations were high in ED visits, and earlier aberrations were also found in ED visits. However, the modified CUSUM method applied to Pearson residuals still caused many false alarms (Figure 5 and Figure 7).

**Figure 8:**
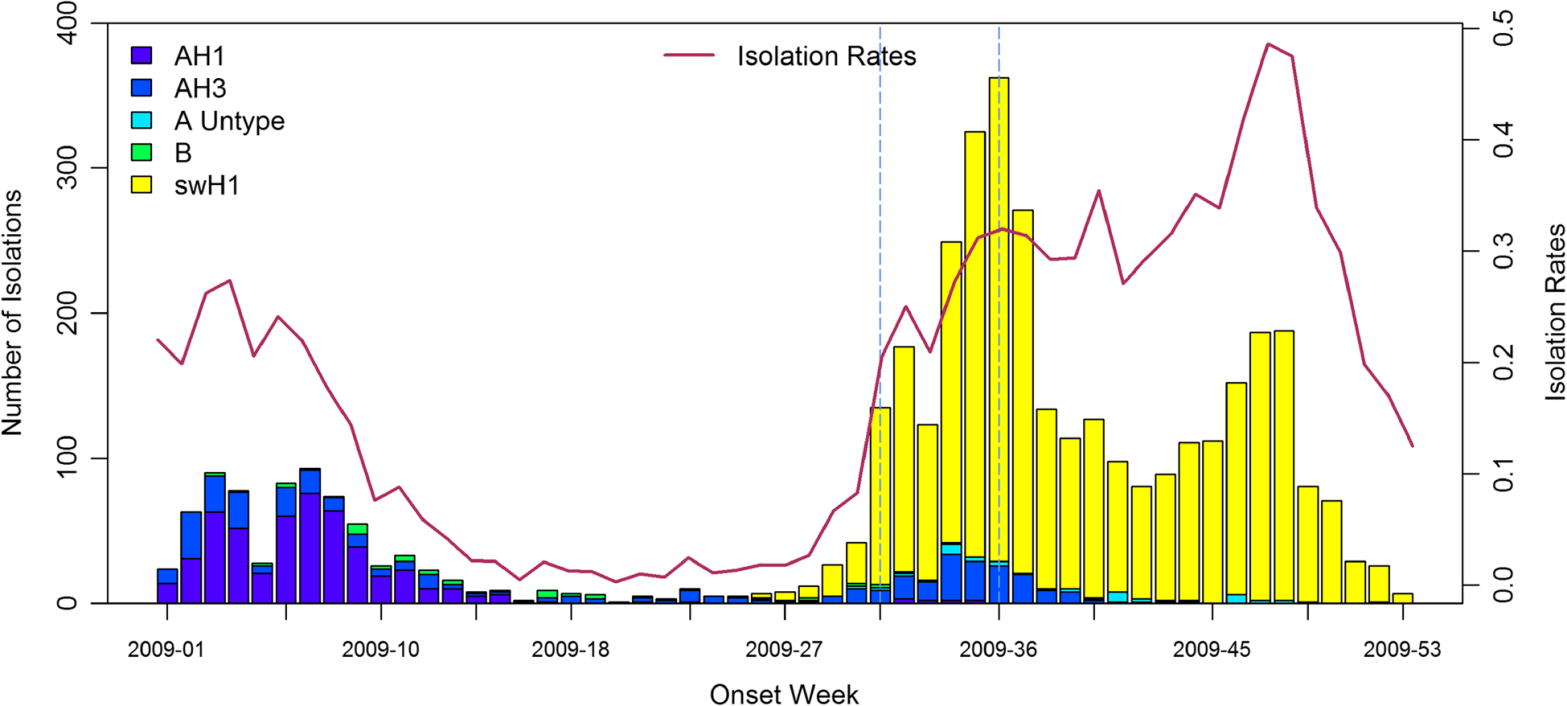
**Weekly National Influenza Isolation Rates in 2009.** *Note: The time period between the two dashed lines was August 2009.

In the first temporal clustering period, the three areas consistently had intensive aberration signals before and after Chinese New Year, when there was also a high virus isolation rate of influenza A/H1 and A/H3 from nationwide virological surveillance (Figure 8). In the second temporal clustering period, there were some single aberrations in early August, before the peak of pandemic H1N1 influenza isolations in the end of August. In the third temporal clustering period (from the end of October to early November), sporadic aberrations in the three areas were detected, and a similar trend was also found in isolation rates (week 46 – week 48). With our proposed SPR method, the first wave of the influenza epidemic, such as before the Chinese New Year or during the pandemic H1N1 influenza outbreak, could be detected by our proposed method and with a lower false alarm rate.

## Discussion

In this study, we proposed using Pearson residuals from fitted negative binomial regression models for aberration detection. The effects of major deterministic and confounding factors associated with ILI epidemics such as temperature, seasons, holidays, the day of the week and temporal dependence were first removed through the regression models. The relatively stationary Pearson residuals of predicted values for current days were then used for aberration detection. This was quite different from the previous studies which monitored ILI visits directly rather than the standardized prediction errors [19]. The approach we proposed in the first stage was like the detrending approach in time series [30]. However, our approach not only removed temporal trends and special events but also adjusted the temperature, which would affect the beginning time and the duration of an ILI epidemic [31]. The variations of residuals of the daily fitted models were often not constant over time. Hence, monitoring residuals for the identification of any aberrations in ILI visits tended to either produce many false alarms, miss true outbreaks, or detect them too late. The Pearson residuals standardized the residuals to make the series more stationary over time. Therefore, the threshold could be fixed at 1.96 for monitoring Pearson residuals for aberration identification with an expected false alarm rate a little higher than 2.5%. The performance is therefore determined mainly by the noise and signal in the observed counts. As demonstrated in the simulation study, when the signal-to-noise ratios were high during influenza season, our method was able to issue warnings in a timely manner after an outbreak. The use of negative binomial models and Pearson residuals for improving the performance of aberration detection has been also considered in the literature [20,21]. These methods are built on cumulative deviations in a traditional framework of control chart for monitoring aberration. Although these methods had great performance in some situations, they may be too technical and complex to be implemented into routine surveillance by general readers. In contrast, the proposed alternative method by monitoring the Pearson residuals directly is simple and relatively easy to use.

The other unique approach used in this study was the definition of ILI cases. We used three criteria to include the cases. The first one was the clinical definition of ILI cases. Although the definition was adopted from ESSENCE [25], it was also effective for our inclusion criteria of ILI cases. Secondly, the first occurrence of ILI cases within 14 days was calculated to avoid counting multiple clinical visits during the same infection course for the same patients. Thirdly, the levels of hospitals which the patients visited were restricted for both outpatient and ED visits. The local living perimeters were the better warning spatial units. Therefore, the outpatient visits were limited to clinics and area hospitals. The ED visits were limited to regional hospitals and area hospitals. In this way, we could keep the data for analysis more representative for the designated study area.

The validation data used for ILI aberration detection were available for much larger regions on a weekly basis for 2009. The daily and small-area virus isolation data were not feasible and were too expensive for the surveillance purpose. The gold standard of exact ILI epidemic time was difficult to obtain. Therefore, we could only use weekly nationwide virus isolation data for external comparison. The exact initial wave of influenza virus isolation in three different areas may have varied. This was a limitation of the present study, so we designed the simulation for evaluating the model’s performance. In 2009, the novel H1N1 influenza began to be isolated in late July 2009. The overall isolation rate surged starting from the first week of August 2009, and reached a peak isolation number in the last week of August 2009. During the same period, the aberrations in both outpatient and ED ILI visits also caused many alerts in early August 2009, which indicated the initial stage of the epidemic. The aberration signals in central and southern Taiwan did not persist too long in August 2009; however, the aberrations of ED ILI visits in northern Taiwan persisted for three weeks.

The data streams in outpatient and ED ILI visits were complementary. The aberration signals were not consistent in the two settings. In some periods, only one setting had the aberration signals; in other periods, both settings had the aberrations together. The example in August 2009 showed that the aberrations were detected a few days earlier in outpatient visits in central Taiwan than in ED visits. However, ED ILI visits were more sensitive for both seasonal and pandemic influenza epidemics than outpatient ILI visits. Previous studies have shown that many countries [32-36] have adopted new, syndromic surveillance systems with data mainly from ED visits. From the viewpoint of a complete surveillance system, we suggest that outpatient visits could also be included for routine ILI surveillance.

Cold temperature had different effects in different areas. Cold and humidity were the two major weather factors correlated to ILI epidemics [23]. However, the temperature was a more significant predictor for ILI visits than relative humidity in this study. A similar observation was also found in another influenza-associated morbidity study [37]. Although Taiwan is located between tropical and sub-tropical areas, the temperature change had different effects on clinic visits for the residents living in the north and the south. Thus, northern Taiwan was especially sensitive to cold temperature. The systematic variables like holidays and weekends were mainly related to outpatients having days off work or school, which caused patients to shift to ED visits. In addition, there were also abnormal surges in medical visits immediately after holidays and weekends. We have found that various covariates had different effects on ILI visits in the three study areas. For applications of the proposed approach to a specific study area, we need to update regression models all the time.

## Conclusions

The seamless surveillance and high coverage rate of health insurance claims data can provide more timely and accurate data than traditional sentinel physician surveillance. By directly monitoring Pearson residuals of fitted negative binomial models to health insurance claims data, near real-time ILI aberration detection in communities is attainable. The successful detection of the 2009 H1N1 pandemic flu in different regions of Taiwan reflected different waves of transmission in August 2009. The complementary signals in both outpatient and ED visits were able to make ILI surveillance more comprehensive. The temporal window for influenza surveillance needs to be adjusted in influenza and non-influenza seasons. Application of the approach to local health insurance claims data can improve the accuracy of outbreak detection in small-area-based ILI surveillance.

## Additional files

Additional file 1:
**Simulated daily counts from negative binomial models with a few additional counts added generated with signal-to-noise ratio equal to 1 on days 601 – 640 (top) and deviation values for monitoring outbreaks for two methods (bottom).** The deviation values of SPR are the Pearson residuals. The deviation values of CUSUM are C3 calculated using the Pearson residuals. The horizontal lines are the threshold of *z*_1 − 0.025_ for SPR and the threshold of 2.88 for the CUSUM. Additional file 2:
**Simulated daily counts from negative binomial models with a few additional counts added generated with signal-to-noise ratio equal to 3 on days 601 – 640 (top) and deviation values for monitoring outbreaks for two methods (bottom).** The deviation values of SPR are the Pearson residuals. The deviation values of CUSUM are C3 calculated using the Pearson residuals. The horizontal lines are the threshold of *z*_1 − 0.025_ for SPR and the threshold of 2.88 for the CUSUM. Additional file 3:
**Simulated daily counts from negative binomial models with a few additional counts added generated with signal-to-noise ratio equal to 5 on days 601 – 640 (top) and deviation values for monitoring outbreaks for two methods (bottom).** The deviation values of SPR are the Pearson residuals. The deviation values of CUSUM are C3 calculated using the Pearson residuals. The horizontal lines are the threshold of *z*_1 − 0.025_ for SPR and the threshold of 2.88 for the CUSUM.

## Abbreviations

ILI: Influenza-like illness
ED: Emergency Department
