# Supplementary material for: Detection of influenza-like illness aberrations by directly monitoring Pearson residuals of fitted negative binomial regression models
Source: BMC Public Health. 2015 Feb 21;15:168. doi: 10.1186/s12889-015-1500-4 (PMC4352259; doi:10.1186/s12889-015-1500-4)

Appendix 3. Simulated daily counts from negative binomial models with a few additional counts added generated with signal-to-noise ratio equal to 5 on days 601 – 640 (top) and deviation values for monitoring outbreaks for two methods (bottom). The deviation values of SPR are the Pearson residuals. The deviation values of CUSUM are C3 calculated using the Pearson residuals. The horizontal lines are the threshold of$z_{1-0.025}$for SPR and the threshold of$3(z_{1-0.025}-1)$for the CUSUM.


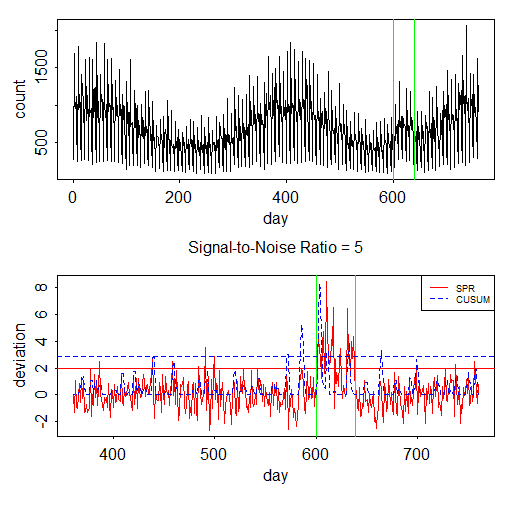

Supplement: Additional file 3: — Simulated daily counts from negative binomial models with a few additional counts added generated with signal-to-noise ratio equal to 5 on days 601 – 640 (top) and deviation values for monitoring outbreaks for two methods (bottom). The deviation values of SPR are the Pearson residuals. The deviation values of CUSUM are C3 calculated using the Pearson residuals. The horizontal lines are the threshold of z 1 − 0.025 for SPR and the threshold of 2.88 for the CUSUM. [file 12889_2015_1500_MOESM3_ESM.docx]
